# Paternal Uniparental Isodisomy of Chromosome 2 in a Patient with *CNGA3*-Associated Autosomal Recessive Achromatopsia

**DOI:** 10.3390/ijms22157842

**Published:** 2021-07-22

**Authors:** Susanne Kohl, Britta Baumann, Francesca Dassie, Anja K. Mayer, Maria Solaki, Peggy Reuter, Laura Kühlewein, Bernd Wissinger, Pietro Maffei

**Affiliations:** 1 Centre for Ophthalmology, Institute for Ophthalmic Research, University Tübingen, 72076 Tübingen, Germany; britta.baumann@med.uni-tuebingen.de (B.B.); anja.ka.mayer@gmail.com (A.K.M.); maria.solaki@med.uni-tuebingen.de (M.S.); peggy.reuter@med.uni-tuebingen.de (P.R.); laura.kuehlewein@med.uni-tuebingen.de (L.K.); bernd.wissinger@med.uni-tuebingen.de (B.W.); 2 Department of Medicine (DIMED), University of Padua, 35121 Padua, Italy; francesca.dassie@unipd.it (F.D.); pietro.maffei@unipd.it (P.M.); 3 Centre for Ophthalmology, University Eye Hospital, University Tübingen, 72076 Tübingen, Germany

**Keywords:** achromatopsia, ACHM, uniparental isodisomy, chromosome 2, *CNGA3*

## Abstract

Achromatopsia (ACHM) is a rare autosomal recessively inherited retinal disease characterized by congenital photophobia, nystagmus, low visual acuity, and absence of color vision. ACHM is genetically heterogeneous and can be caused by biallelic mutations in the genes *CNGA3*, *CNGB3*, *GNAT2*, *PDE6C*, *PDE6H*, or *ATF6*. We undertook molecular genetic analysis in a single female patient with a clinical diagnosis of ACHM and identified the homozygous variant c.778G>C;p.(D260H) in the *CNGA3* gene. While segregation analysis in the father, as expected, identified the *CNGA3* variant in a heterozygous state, it could not be displayed in the mother. Microsatellite marker analysis provided evidence that the homozygosity of the *CNGA3* variant is due to partial or complete paternal uniparental isodisomy (UPD) of chromosome 2 in the patient. Apart from the ACHM phenotype, the patient was clinically unsuspicious and healthy. This is one of few examples proving UPD as the underlying mechanism for the clinical manifestation of a recessive mutation in a patient with inherited retinal disease. It also highlights the importance of segregation analysis in both parents of a given patient or especially in cases of homozygous recessive mutations, as UPD has significant implications for genetic counseling with a very low recurrence risk assessment in such families.

## 1. Introduction

Achromatopsia (ACHM; synonymous with rod monochromatism; ACHM2, MIM 216900; ACHM3, MIM 262300; ACHM4, MIM 613856; ACHM5/COD4, MIM 613093; ACHM6/RCD3A, MIM 610024; and ACHM7, IM616517) is a rare, autosomal recessive disorder with an estimated prevalence of 1 in 30,000 to 1:50,000. It is a congenital retinal disease affecting only the cone photoreceptor, and photophobia and nystagmus are the first symptoms manifesting within the first weeks or months of life. Due to lack of cone photoreceptor function, ACHM is characterized by color blindness and low visual acuity (20/400–20/200 Snellen equivalent). ACHM was believed to be a stationary disease, but recent studies by optical coherence tomography have shown that there is progressive degeneration of the central retina and cone photoreceptors [1,2,3,4,5].

ACHM is genetically heterogeneous and known to be caused by mutations in six genes: *CNGA3*, *CNGB3*, *GNAT2*, *PDE6C*, *PDE6H*, and *ATF6* [6,7,8,9,10,11,12,13]. The first five genes encode important functional components of the cone phototransduction cascade (G-protein transducin GNAT2 (guanine nucleotide-binding protein G(t) subunit alpha 2; MIM 139340); cone-photoreceptor phosphodiesterase catalytic subunit PDE6C (cone cyclic GMP-specific 3′,5′ cyclic phosphodiesterase alpha-prime; MIM 600827) and inhibitory subunit PDE6H (cone cyclic GMP-specific phosphodiesterase gamma; MIM 601190); cone photoreceptor cyclic nucleotide gated cation (CNG) channel CNGA3 (cyclic nucleotide-gated cation channel alpha 3; MIM 600053) and CNGB3 (cyclic nucleotide-gated cation channel beta 3; MIM 605080)), while the ubiquitously expressed transcription factor ATF6 (activating transcription factor 6; MIM 605537) is known for its function in the unfolded protein response and endoplasmic reticulum stress response [14,15].

In Europe and the United States, biallelic mutations in *CNGB3* are the major cause of ACHM, accounting for 50–60% of cases, especially due to a common founder mutation c.1148delC, followed by biallelic mutations in *CNGA3*, which are found in 30% of ACHM patients in these populations [16,17]. In contrast, biallelic mutations in *CNGA3* account for the majority of ACHM patients of Chinese (80%) and Israeli/Palestinian (84%) origin [18,19]. The other ACHM-associated genes play a minor role, being responsible for only 0.5–3% of cases [16].

*CNGA3*- and *CNGB3*-associated ACHM are in the focus, as several AAV-based gene supplementation therapy trials are ongoing (NCT02610582, NCT03758404, NCT02935517, NCT02599922), and one trial has already been successfully finished and published [20].

## 2. Results

An Italian female patient with a clinical diagnosis of ACHM without family history of this condition was referred to the Centre for Ophthalmology Tübingen for genetic testing in a research setting. The patient suffered from photophobia since birth and was diagnosed to have ACHM at the age of 10 years. Her visual acuity at the age of 18 was 20/200 Snellen equivalent with a correction of +3.00 diopters in both eyes. The patient exhibited a fine pendular nystagmus that was more pronounced when looking at distant targets. Intraocular pressure was within normal limits in both eyes (14 mmHg). Anterior and posterior segment examination was unremarkable except for the margins of the optic discs that were not absolutely sharp. Full-field electroretinogram showed symmetrical severely reduced cone responses and abnormalities in the rod responses. Dark adaptation curves were typical for ACHM. Color vision testing using a Panel D-15 test and a Nagel anomaloscope exhibited findings typical for ACHM. Kinetic visual field testing showed normal outer visual field boundaries.

**Figure 1:**
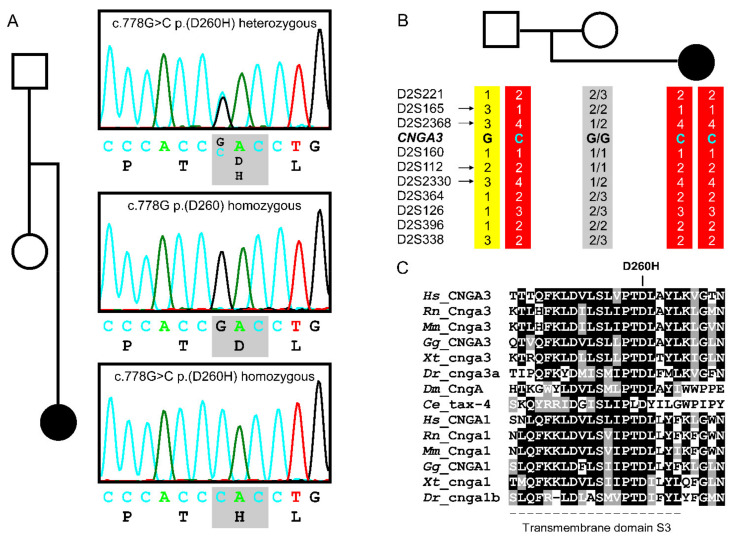
Segregation analysis, haplotype reconstruction, and conservation of *CNGA3* c.778G>C;p.(D260H). (**A**) Segregation analysis via Sanger sequencing for *CNGA3* exon 7 in family CHRO978, as shown by the electropherograms obtained from both parents and the index patient. The father is heterozygous for the variant c.778G>C (top), while the mother does not carry this variant (middle). The affected daughter is homozygous for this variant in *CNGA3* (bottom). (**B**) Haplotype reconstruction of chromosome 2. Microsatellite marker analysis in the patient and both parents demonstrated paternal uniparental isodisomy of chromosome 2, either fully or partially. The patient inherited two copies (or partial chromosomal segments) of the paternal chromosome (red haplotype) carrying and flanking the *CNGA3* variant c.778G>G;p.(D260H), while the maternal chromosome was not or only partially transmitted. Informative markers are marked by an arrow. Of note, the two haplotypes of the mother could not be reconstructed. (**C**) Conservation of the mutated amino acid residue *CNGA3* p.D260H among CNGA3 and CNGA1 polypeptides across various species. Protein sequence alignment was performed using Clustal Omega (https://www.ebi.ac.uk/Tools/msa/clustalo/ (accessed on 16 June 2021)) and BoxShade (https://embnet.vital-it.ch/software/BOX_form.html (accessed on 16 June 2021)). Reference sequences from NCBI (https://www.ncbi.nlm.nih.gov/ (accessed on 16 June 2021)): CNGA3: *H. sapiens* NP_001289.1, *R. norvegicus* NP_445947.1, *M. musculus* NP_001268939.1, *G. gallus* NP_990552.1, *X. tropicalis* XP_031752880.1, *D. rerio* XP_005166141.1, *D. melanogaster* NP_477116.1, *C. elegans* NP_499033.1, CNGA1: *H. sapiens* NP_001366199.1, *R. norvegicus* NP_445949.2, *M. musculus* NP_031749.2, *G. gallus* NP_990551.1, *X. tropicalis* XP_017950934.1, *D. rerio* XP_701036.4.

Genetic research testing revealed an apparent homozygous missense variant c.778G>C;p.(D260H) in exon 7 of *CNGA3*. The variant has not been reported in the literature, various mutation databases (i.e., HGMD^®^—Human Gene Mutation Database, ClinVar https://www.ncbi.nlm.nih.gov/clinvar (accessed on 16 June 2021), LOVD^3^—Leiden Open Variation Database https://www.LOVD.nl/CNGA3 (accessed on 16 June 2021)), nor has it been observed in population databases (i.e., gnomAD browser; https://gnomad.broadinstitute.org/gene/ENSG00000144191 (accessed on 16 June 2021)). The variant affects an amino acid residue in transmembrane domain S3 of the CNGA3 polypeptide. This amino acid residue is conserved not only in all CNGA3 orthologs but also in rod-photoreceptor-specific CNGA1 paralogs (Figure 1C).

Segregation analysis by PCR and Sanger sequencing proved the father to be a heterozygous carrier, while the variant could not be detected in the mother (Figure 1A). A heterozygous deletion in the mother explaining the apparent homozygosity of the mutation in the patient was ruled out by long-distance PCR and a SYBR Green-based quantitative real-time PCR copy number assay. Segregation analysis and haplotype reconstruction for ten microsatellite markers covering the entire chromosome 2 in the patient and both parents revealed homozygosity in the patient for all tested markers for the paternal allele, providing evidence for complete (or partial) paternal uniparental isodisomy (UPD) of chromosome 2 in the patient (Figure 1B).

**Figure 2:**
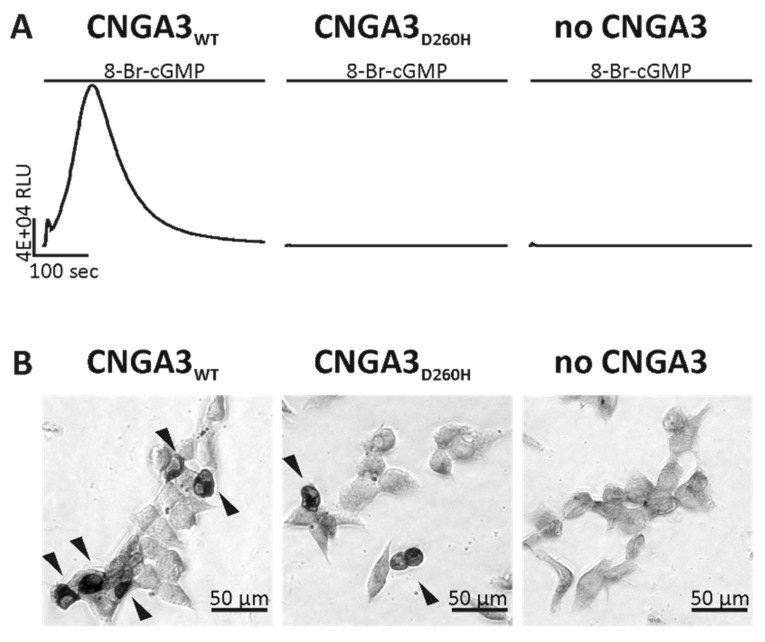
Aequorin-mediated luminescence signals and immunocytochemical staining of wild-type (WT) and mutant (D260H) CNGA3 channels. (**A**) The luminescence signal of HEK293 cells either expressing aequorin and CNGA3_WT_ (left) or aequorin and CNGA3_D260H_ (middle) or aequorin only (right) was recorded for a duration of 400 s. Application of the CNGA3 channel ligand 8-bromoguanosine-3′,5’-cyclic monophosphate (8-Br-cGMP) 6 s after starting the recording resulted in an increase of the luminescence signal only in cells expressing CNGA3_WT_. HEK293 cells expressing mutant CNGA3_D260H_ displayed no luminescence signal classifying the variant as non-functional. Cells expressing aequorin-only served as a negative control. RLU: relative light units. (**B)** Detection of CNGA3 protein expression was conducted by immunocytochemical diaminobenzidine staining. HEK293 cells expressing the CNGA3 channel protein are marked with an arrow head. Expression of the channel mutant CNGA3_D260H_ in HEK293 cells could be confirmed (middle). CNGA3_WT_-expressing cells (left) and cells expressing no CNGA3 channel (right) served as a positive and negative controls, respectively.

The variant is predicted to be disease-causing by various tools (i.e., SIFT, PolyPhen, Provean) and classified as likely pathogenic according to the ACMG (American College of Medical Genetics) guidelines. In vitro heterologous expression and analysis via a bioluminescent calcium reporter assay of the CNGA3_D260H_ mutant versus wild-type CNGA3 in HEK293 (human embryonic kidney) cells provided evidence that this variant results in complete loss of channel function (Figure 2A). Similarly, another amino acid substitution at the very same amino acid residue—c.778G>A;p.(D260N)—had previously been shown to result in complete channel function loss in in vitro studies [21,22]. Loss of CNGA3 or CNGA3 function cannot be compensated, as *CNGA3* encodes for the channel forming A subunit of the cone photoreceptor CNG channel. Using immunocytochemistry, we were able to confirm the expression of the channel mutant CNGA3_D260H_ in transfected HEK293 cells despite the fact that a calcium influx was not detectable in the aequorin-based bioassay (Figure 2B).

Upon this finding, we initiated a general clinical investigation and family history of the patient when she was 49 years old. Her paternal grandmother had dextrocardia and supernumerary kidneys with end-stage kidney failure on dialytic therapy. The mother underwent nephrectomy for kidney cancer. There was no parental consanguinity, nor were any visual disturbances reported among family members. The patient had a regular growth for weight and height, as well as age of menarche and menstrual cycles. She reported allergy to house dust and a history of migraines that was treated with triptans as needed. She suffered from photophobia and nystagmus since the first weeks of life. At the age of 10, the patient was diagnosed with ACHM. The visual disturbances remained stable over the years and the patient was still independent in daily living activities. On physical examination, the anthropometric parameters were within normal limits (weight 45.4 kg; height 153 cm; BMI 19.2). No significant dysmorphisms of the head or arms were observed. She had a normal blood pressure (120/70 mmHg) and heart rate (76 m’). The clinical examination of the neck, chest, heart, abdomen, and neurological system were unremarkable. A general laboratory investigation including white blood cell count, platelet count test, hemoglobin, glycaemia and lipid profile, thyroid-stimulating hormone, electrolytes, and kidney and liver function tests was unremarkable. Instrumental investigations by chest X-ray, electrocardiography, echocardiography, and thyroid and abdominal ultrasound showed multiple bilateral kidney cysts with a maximum diameter of 14 mm.

## 3. Discussion

Uniparental isodisomy can be disease-causing due to disrupted imprinting or unmasking of recessive mutations. The patient in this study presented with typical symptoms of autosomal recessive ACHM, and a homozygous novel missense variant c.778G>G; p.(D260H) in *CNGA3* was identified. Functional analysis showed that this variant results in complete function loss of heterologously expressed mutant CNG channels indicating that this missense variant is a true pathogenic mutation. Segregation analysis and haplotype reconstruction provided evidence for complete or partial paternal UPD of chromosome 2, thereby unmasking the *CNGA3* mutation as the cause of the cone photoreceptor disease due to inheritance of two identical chromosomes (or partial chromosomal segments) from the father, while the maternal chromosome (or partial chromosomal segments) was lost.

Uniparental isodisomy was thought to be a rare phenomenon, but recent data suggest that it may be twice as common as previously expected, with a prevalence of approximately 1 in 2000 births [23].

While it is disease-relevant in imprinting disease, there is often no disease association—except for manifesting recessive mutations. A comprehensive literature search showed that several cases of UPD of chromosome 2 have been reported, some reflecting a similar situation as in our patient, where a recessive mutation manifests in the patient due to the UPD by inheriting two copies of a recessive mutation by one parent [24,25,26], but also generally healthy individuals have been identified by a genome-wide SNP scan or parentage testing [27,28,29]. Several genes have been predicted (http://www.geneimprint.com/site/genes-by-species (accessed on 16 June 2021); i.e., *OTX1*, *VAX2*, *CYP1B1*) or experimentally shown to be imprinted on chromosome 2 (i.e., *LRRTM1*, *GPR1*, *ZDBF2*), but none of these have been shown to be associated with a disease caused by imprinting (https://www.omim.org/ (accessed on 16 June 2021)). They rather result in disease due to classical point mutations (e.g., mutations in *CYP1B1* result in glaucoma [26], mutations in *ABCG8* are associated with sitosterolemia [30]), or knockout animals have been shown to develop a phenotype (i.e., *Otx1* knockout mice show epileptic behavior [31], *Vax2* knockout results in abnormal retinal and optic nerve development [32], and *Zfp36l2* knockout is embryonically lethal [33]). These findings are in line with the fact that our patient is of general good health apart from the isolated retinal phenotype of ACHM.

It has been suggested that UPD is associated with an older maternal age at conception or an advanced parental age in general [34,35]. This does not hold true for our patient, as the parents were in their early and mid-twenties when the patient was born.

The herein presented case is one of very few examples for this mechanism observed and described in inherited retinal dystrophy [36,37,38,39,40], including UPD of chromosome 2 in a patient with a homozygous mutation in *MERTK* [41], and the first for ACHM.

## 4. Materials and Methods

The study was conducted in accordance with the Declaration of Helsinki, with approval from the ethics committee of the University of Tübingen (project nos. 349/2003V and 116/2015BO2). Venous blood samples of the index patient and both parents were collected upon written informed consent.

Ophthalmological examinations included visual acuity testing, intraocular pressure measurement, anterior and posterior segment examination, full-field electroretinography, dark adaptation curves, color vision testing using a Panel D-15 test and a Nagel anomaloscope, and kinetic visual field testing.

Patient’s medications; allergies; and past medical, surgical, social, and family history were reviewed. Anthropometric measurements were taken with the patient wearing only light clothes without shoes. Height was measured to the nearest 0.01 m using a stadiometer. Body weight was determined to the nearest 0.1 kg using a calibrated balance beam scale. Body mass index was calculated as kg/m^2^, where kg is the patient’s weight in kilograms and m^2^ is the height in meters squared. Blood pressure and heart rate were recorded after 10 min rest at the brachial artery of the dominant arm using a validated semi-automated oscillometric device in the seated position. A thorough and systematic clinical examination was done. Complete blood count, protein electrophoresis, serum lipids, renal and liver function, blood glucose and HbA1c levels, electrolytes, thyroid function, and urine analysis were measured by standard laboratory procedures. Instrumental investigations included chest X-ray, electrocardiography, echocardiography, and thyroid and abdominal ultrasound.

Sanger sequencing of all coding exons of *CNGA3* on an ABI3130 capillary sequencer (Applied Biosystems, Thermo Fisher Scientific, Waltham, MA, USA) was performed on PCR-amplified genomic DNA obtained from venous blood as previously described [7,21]. Segregation analysis was also performed by PCR and Sanger sequencing (Figure 1A). In addition, the common ACHM-associated variant *CNGB3* c.1148del was excluded.

The *CNGA3* variant was classified according to the standards and guidelines provided by the American College of Medical Genetics and Genomics (ACMG) and the Association for Molecular Pathology (AMP) [42]. The potential impact and pathogenicity of the missense change was further assessed by applying various prediction tools embedded in Alamut Visual software (Interactive Biosoftware, Rouen, France), literature search, and conservation between various CNGA3 and CNGA1 orthologs and paralogs. The variant was annotated according to NCBI reference sequence for *CNGA3* (NM_001298.3, ENST00000272602.7, GRCh38 genome assembly) comprising seven coding exons.

To test for a putative heterozygous deletion of *CNGA3* exon 7 in the mother and the patient, we performed a custom-designed amplicon-based assay for *CNGA3* exon 7 applying the QuantiTect SYBR Green PCR Kit (QIAGEN, Hilden, Germany), primers 5′-CTCAAGAGCCTCCCAGACAA-3′ and 5′-AGCTTCAGCACCAGCTCCA-3′ and *SDC4* as reference gene, as previously described for *CNGB3* [16].

For human chromosome 2 segregation analysis, ten microsatellite markers (D2S2211, D2S165, D2S2368, D2S160, D2S112, D2S2330, D2S364, D2S126, D2S396, D2S338) covering the entire chromosome 2 were selected from the ABI PRISM^®^ Linkage Mapping Set (Applied Biosystems, Thermo Fisher Scientific) and genotyped by PCR amplification and fragment sizing on an ABI3130 capillary sequencer (Applied Biosystems, Thermo Fisher Scientific) in Gene Scan mode. Microsatellite marker typing is presented in Supplementary Figures S1 and S2, Table S1.

Wild-type and p.D260H mutant CNGA3 channel functionality and surface expression were evaluated in transiently transfected human embryonic kidney (HEK293) cells using an aequorin-based luminescence bioassay. The generation of the wild-type human CNGA3 expression construct was described previously [43]. In vitro mutagenesis PCR was performed to generate the mutant CNGA3 expression construct CNGA3_D260H_. The establishment of an expression construct for cytosolic apo-aequorin was described previously [44]. HEK293 cells (DSMZ-German Collection of Microorganisms and Cell Cultures GmbH, Braunschweig, Germany) were cultured in Dulbecco’s modified Eagle’s medium (Thermo Fisher Scientific) supplemented with 10% fetal calf serum (Thermo Fisher Scientific), 1% penicillin/streptomycin (Thermo Fisher Scientific), and 1% amphotericin B (Pan Biotech, Aidenbach, Germany) at 5% CO_2_ at 37 °C. HEK293 cells were seeded at a density of 3.5 × 10^5^ cells per 24 well and transfected on the next day with 1 µg wild-type or mutant CNGA3 and 1.5 µg of apo-aequorin expression plasmid using Lipofectamine™ 2000 (Thermo Fisher Scientific) following the manufacturer’s instructions [44]. Control cells were transfected only with 2 µg apo-aequorin expression plasmid. Six hours after transfection start, cells were re-plated in a white opaque 96 well plate. After overnight incubation, transfected cells were treated with 3 mM sodium butyrate (Sigma-Aldrich, St Louis, MO, USA) for 24 h to enhance protein expression. Prior to the luminescence assay, cells were washed with calcium imaging buffer (CI; 150 mM NaCl, 5 mM KCl, 2 mM CaCl_2_, 2 mM, MgCl_2_, 10 mM HEPES (pH 7.4), 30 mM glucose) and incubated with 8 µM coelenterazine (Biomol GmbH, Hamburg, Germany) in CI for 4 h at 37 °C to reconstitute the apo-aequorin. Following washing with CI and CI-high Ca^2+^ (CI buffer with 10 mM Ca^2+^), 190 µL of CI-high Ca^2+^ per 96 well was added for the subsequent luminescence bioassay. Measurements were performed with the LUMIstar^®^ Omega luminescence plate reader (BMG Labtech GmbH, Ortenberg, Germany) at 37 °C. Luminescence was detected 6 s before and 394 s after automatic application of 10 µL 100 mM 8-bromoguanosine-3′,5’-cyclic monophosphate (8-Br-cGMP; BIOLOG Life Science Institute, Bremen, Germany)—a membrane-permeable CNG channel agonist. Samples were measured in triplicate, and three independent transfections were performed.

CNGA3 protein expression was verified by immunocytochemistry. HEK293 cells were seeded at a density of 3.5 × 10^5^ cells per 24 well and transfected on the next day, as described above using 2 µg wild-type or mutant CNGA3 expression construct. Following treatment with 3 mM sodium butyrate for 24 h, immunocytochemical staining was performed as previously described [45]. CNGA3 was detected using the custom primary antibody SA3899 and the horseradish peroxide-coupled goat anti-rabbit antibody (Merck KGaA, Darmstadt, Germany). For visualization, we performed a diaminobenzidine (DAB; Sigma Aldrich GmbH, Munich, Germany) staining, applying 0.005% DAB and 0.12% hydrogen peroxide in phosphate-buffered saline.

## 5. Conclusions

We herein report a unique case of ACHM due to UPD of chromosome 2. UPD is a rare phenomenon due to inheritance of both chromatids of a single chromosome from one parent—here, the paternal chromosome 2, while the maternal chromosome was not transmitted or lost from the zygote. UPD thus uncovered a recessive mutation present in just one parent—here, the missense variant c.778G > C; p.(D260H) in *CNGA3* in the father, causing ACHM in the index patient. It is one of very few examples for this mechanism observed and described in inherited retinal dystrophy and the first for ACHM. The detection of UPD highlights the importance of segregation analysis and has significant implications for genetic counseling with a very low recurrency risk assessment in such families.

## Data Availability

Any data presented in this study are available on request from the corresponding author.

## Supplementary Materials

The following are available online at https://www.mdpi.com/article/10.3390/ijms22157842/s1.
