# Supplementary material for: Paternal Uniparental Isodisomy of Chromosome 2 in a Patient with CNGA3-Associated Autosomal Recessive Achromatopsia
Source: Int J Mol Sci. 2021 Jul 22;22(15):7842. doi: 10.3390/ijms22157842 (PMC8346044; doi:10.3390/ijms22157842)
Supplement: Supplementary file 1 [file ijms-22-07842-s001.zip › ijms-1282533-supplementary.pdf]

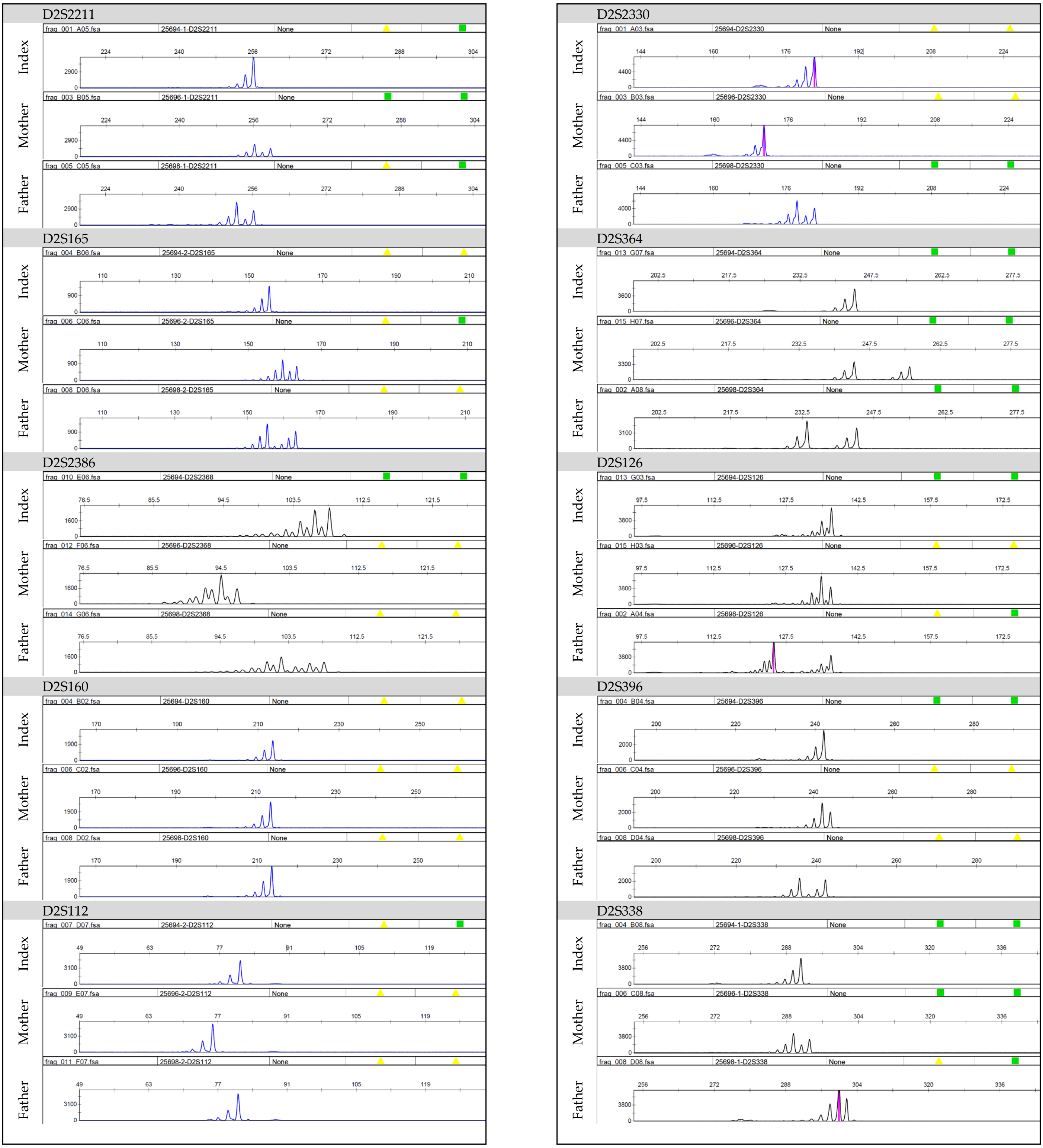

Supplementary Figure S1: Microsatellite marker fragment sizing for human chromosome 2.

Supplementary Table S1: Microsatellite marker typing for human chromosome 2.

	D2S2211		D2S165		D2S2386		D2S160		D2S112	
	**Allele 1**	**Allele 2**	**Allele 1**	**Allele 2**	**Allele 1**	**Allele 2**	**Allele 1**	**Allele 2**	**Allele 1**	**Allele 2**
#25694 Index	256	256	155	155	108	108	213	213	81	81
#25696 Mother	256	259	159	163	94	96	213	213	75	75
#26968 Father	252	256	155	163	102	108	213	213	81	81
	uninformative						uninformative			

	D2S2330		D2S364		D2S126		D2S396		D2S338	
	**Allele 1**	**Allele 2**	**Allele 1**	**Allele 2**	**Allele 1**	**Allele 2**	**Allele 1**	**Allele 2**	**Allele 1**	**Allele 2**
#25694 Index	182	182	244	244	136	136	242	242	291	291
#25696 Mother	168	170	244	255	134	138	242	244	289	293
#26968 Father	178	182	233	244	124	136	235	242	291	293
			uninformative		uninformative		uninformative			

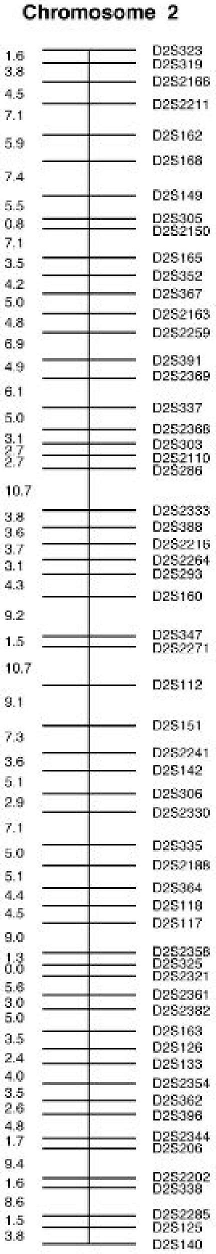

Supplementary Figure S2: Microsatellite markers and physical distances of the ABI PRISM® Linkage Mapping Set (Thermo Fisher Scientific; https://genome.med.harvard.edu/documents/genotyping/Human_Linkage.pdf).
